# Computer Vision-Based Structural Displacement Measurement Robust to Light-Induced Image Degradation for In-Service Bridges

**DOI:** 10.3390/s17102317

**Published:** 2017-10-11

**Authors:** Junhwa Lee, Kyoung-Chan Lee, Soojin Cho, Sung-Han Sim

**Affiliations:** 1 School of Urban and Environmental Engineering, Ulsan National Institute of Science and Technology (UNIST), Ulsan 44919, Korea; lee.junhwa@unist.ac.kr; 2 Korea Railroad Research Institute, Uiwang 16105, Korea; kclee@krri.re.kr; 3 Department of Civil Engineering, University of Seoul, Seoul 02504, Korea

**Keywords:** adaptive ROI, computer vision, displacement

## Abstract

The displacement responses of a civil engineering structure can provide important information regarding structural behaviors that help in assessing safety and serviceability. A displacement measurement using conventional devices, such as the linear variable differential transformer (LVDT), is challenging owing to issues related to inconvenient sensor installation that often requires additional temporary structures. A promising alternative is offered by computer vision, which typically provides a low-cost and non-contact displacement measurement that converts the movement of an object, mostly an attached marker, in the captured images into structural displacement. However, there is limited research on addressing light-induced measurement error caused by the inevitable sunlight in field-testing conditions. This study presents a computer vision-based displacement measurement approach tailored to a field-testing environment with enhanced robustness to strong sunlight. An image-processing algorithm with an adaptive region-of-interest (ROI) is proposed to reliably determine a marker’s location even when the marker is indistinct due to unfavorable light. The performance of the proposed system is experimentally validated in both laboratory-scale and field experiments.

## 1. Introduction

Structural health monitoring (SHM) is an essential tool for the effective maintenance of civil infrastructure, with a number of SHM systems employed in real-world applications [1,2,3,4,5]. Data acquisition of structural responses is a fundamental step in SHM systems where the data is subsequently processed for condition assessment and decision-making. Displacement responses from a civil engineering structure are considered to be informative in evaluating the structure’s current structural condition and safety. As it is directly related to structural stiffness and loadings, displacement can be an indicator of structural changes and excessive external loadings [6,7]. For example, the plastic deformation ratios of building structures are estimated by drift displacement data [8]. Most design codes used in modern countries (e.g., the AASHTO LRFD bridge design specification) specify maximum displacement levels for bridge structures to assure structural safety and usability. Thus, displacement information is commonly employed for infrastructure maintenance purposes.

Displacement sensors, such as linear variable differential transformers (LVDT) and strain-based displacement transducers, are widely adopted for conducting displacement measurements in practice. These sensors are typically placed between a target point on a structure and a fixed reference point, measuring relative displacements. A sensor’s installation requires additional supporting structures with respect to the fixed references that are often unavailable or difficult to prepare in field testing involving full-scale civil engineering structures. Furthermore, vibrations of the supporting structures can significantly degrade measurement accuracy. Thus, using traditional sensors to measure displacement responses from a full-scale structure is considered to be inefficient.

Recent research efforts have focused on effectively addressing this issue and providing a practical means for displacement measurement. Specifically, extant research includes the following: (1) the development of indirect displacement estimation algorithms to convert other physical quantities, such as acceleration and strain, to displacement; and (2) an applicability investigation of relatively new sensors, including the laser Doppler vibrometer (LDV), global positioning systems (GPS), and computer vision-based approaches. Indirect estimation methods typically use acceleration and strain measurements that are independent of the reference points [9,10,11,12,13,14,15,16,17,18,19]. However, the estimation performance and accuracy of the indirect methods are highly dependent on displacement conversion algorithms that should be carefully handled to avoid unexpected large errors. The laser Doppler vibrometer (LDV) is a representative noncontact-type sensor that measures displacement by the Doppler shift of emitted and reflected laser rays [20,21]. Although the LDV exhibits excellent accuracy, disadvantages such as high cost and a limitation wherein an LDV only can measure displacement in the direction of the emitted laser have prevented the widespread adoption of the LDV in practice. Several studies have investigated global positioning systems (GPS) with respect to structural health monitoring (SHM) as summarized succinctly by Im et al. [22]. The GPS is an attractive and promising alternative for deflection monitoring. However, the positioning accuracy of current GPS technology is considered as only appropriate for structures with large deflections, such as long-span bridges [23,24,25] and high-rise buildings [26], while most of the other civil structures with small deflections require better alternatives [27].

Previous studies have reported that computer vision-based methods possess the potential to address the issues in existing techniques [28,29,30,31,32,33,34,35,36,37,38,39,40,41,42,43,44,45,46,47,48,49,50,51,52,53]. The existing vision-based methods differ by (1) non-target approaches, (2) feature detection, and (3) coordinate transforms. The non-target approaches utilize noticeable features from a structure, which are tracked to measure displacement. Example algorithms include orientation code matching (OCM) [28,29], Kanade–Lucas–Tomasi (KLT) [30], Eulerian-based algorithms [31,32], and upsampled cross correlation (UCC) [33]. Target-based approaches use a target marker with specially designed features, such as a circle [34,35,36,37,38,39], a checkerboard [40,41,42,43,44], or a random pattern [45]. Once a feature is detected, the position of the feature is transformed to the physical domain by using a coordinate transform. Several different transformation methods have been employed, such as simple scaling [28,29,30,31,32,33,34,35,40,44], the affine transform [36,37], extrinsic parameters acquisition [42,43], and the homography transform [46,47,48,49,50]. Previous studies have shown the immense potential of computer vision for displacement sensing and other SHM applications, such as system identification [51] and long-span bridge displacement measurement [52].

Several practical issues in computer vision-based displacement sensing have been identified in the literature, including the use of target markers, the selection of camera locations, and light-induced error. The non-target approaches are convenient in that they do not need an installation of target markers. Despite the convenience, target-based measurement becomes useful when combined with the homography transform, which can greatly increase field applicability by allowing cameras to be arbitrarily placed [46,47,48,49,50]. Regarding light-induced error, few studies have examined feature detection in a harsh field-testing environment, particularly those with adverse light conditions [53]. Sunlight causes an image blur of target markers and thereby leads to significant error in finding features in the captured images.

This study presents a computer vision-based approach for displacement measurement tailored to field testing for civil engineering structures. Following the hardware configuration and coordinate transform used in previous studies, the proposed approach includes an image-processing scheme associated with an adaptive region-of-interest (ROI) process to reliably identify marker locations under the presence of light-induced image degradation. A laboratory-scale experiment is conducted to validate the proposed method in terms of light conditions. Field-testing results involving a 40-m-long steel box girder bridge are presented to validate the performance of the proposed approach.

## 2. Computer Vision-Based Displacement Measurement

### 2.1. Overview

**Figure 1:**
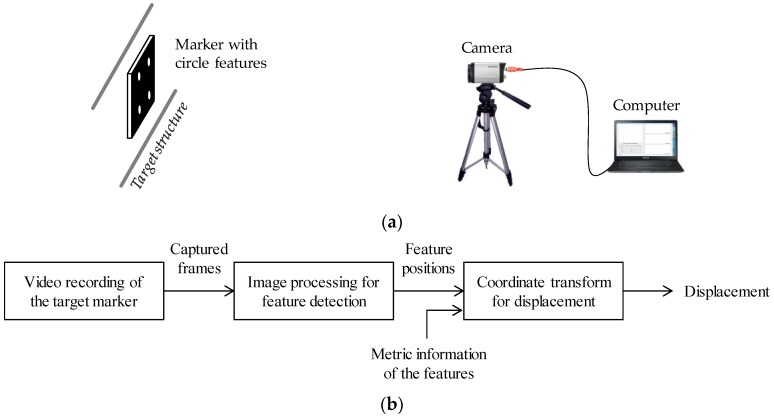
Common configuration of vision-based displacement measurement approaches: (**a**) hardware; (**b**) software.

Computer vision-based displacement measurement methods typically consist of hardware and software components (see Figure 1). The hardware part can be prepared with a commercial camera, a computer for data acquisition and processing, and a user-defined target marker to build a highly cost-effective system. The marker’s movements are recorded by the camera and simultaneously transferred to the computer that calculates the displacement using image-processing algorithms and coordinate transforms.

As the coordinate transforms relate the image and the physical coordinates, the marker and the camera must be properly aligned and placed considering the limitations of the coordinate transforms selected. However, to find an appropriate location where the camera can be securely placed and the assumptions in the coordinate transforms are not violated is often challenging in field testing. In this section, coordinate transforms introduced by the previous research works are briefly described to discuss issues in camera placement.

**Figure 2:**
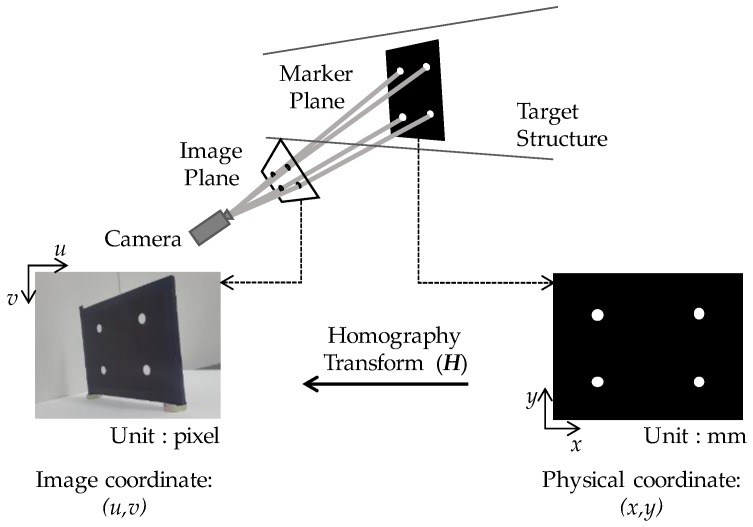
Illustration of the homography transform between the image and marker planes.

Four types of coordinate transforms are employed in the existing vision-based methods, which are (1) simple scaling [28,29,30,31,32,33,34,35,40,44], the affine transform [36,37], the extrinsic parameters acquisition method [42,43], and the homography transform [46,47,48,49,50]. Simple scaling multiplies the scaling factor (unit: mm/pixel) to the measured image coordinate displacement; thus, the direction of the target’s movement and the image displacement must be aligned with each other. The affine transform requires the camera and the target marker to be aligned perpendicular to each other, because the assumptions adopted in defining the transform disregard the perspective projection. Extrinsic camera parameters, which describe six degree-of-freedom motions (three-dimensional (3D) translation and 3D rotation) of the target marker, can be acquired only with a short focal length lens [54], which limits the camera to stay near the target marker. The homography transform can map the image plane to the marker plane regardless of the camera’s position as Figure 2 describes. As a result, the homography transform is regarded as an appropriate solution for unconstrained camera positioning in field testing, and is used in this study.

The displacement calculation using planar homography is based on the relationship between the physical coordinates in the marker plane and the image coordinates in the captured image as described in Equation (1).

(1) $$si_{3\times 1}=H_{3\times 3}w_{3\times 1}$$

where *s* denotes a scaling factor, ***i***_3×1_ = [*u v* 1]^T^ denotes an image coordinate, ***H***_3×3_ denotes the homography matrix, and ***w***_3×1_ = [*x y* 1]^T^ denotes a physical coordinate. Firstly, the homography matrix is computed by means of the direct linear transformation (DLT) algorithm [52], which adopts at least four physical coordinates in the marker plane and the corresponding image coordinates in the first image to determine the optimal transformation matrix between the image and physical coordinate systems. Once the homography matrix is computed from the first image, a time history of the marker’s movement is calculated by the inverse homography transform of the image coordinates in the sequentially acquired images into the physical coordinates.

In full-scale civil structure applications, the vision-based approaches meet several practical issues, such as the selection of a camera installation point and light-induced error. In the case of the camera installation issue, the homography transform can be a solution allowing a camera to be placed at an arbitrary point. The light conditions in a field-testing environment can have an adverse effect on the captured images for a displacement measurement. To enhance the field applicability of the computer vision-based method, this paper focuses on addressing light-induced image degradation, while utilizing the homography transform to provide a wide freedom of camera installation.

### 2.2. Light-Induced Image Degradation

The features on a target marker could be inaccurately detected under adverse light conditions, particularly in field testing. Direct sunlight or reflected light can cause significant degradation in images captured with target markers. Such degradation can cause imbalanced brightness, a loss of definite edges, or a change in feature shapes, which results in the erroneous positioning of features. Feng and Feng [53] addressed this issue by providing a lab-scale experiment conducted in a dim-light condition. However, the effects of excessive light exposure need to be further investigated. In this section, the action of adverse light on the feature detection process is briefly described with an example.

**Figure 3:**
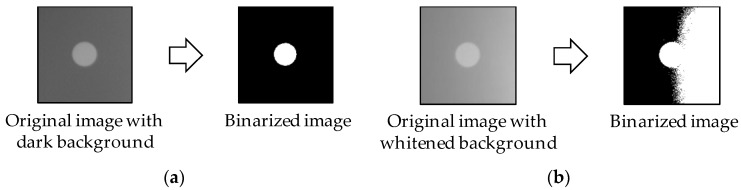
Image binarization using the Otsu threshold [55] for feature detection: (**a**) clear image; (**b**) degraded image with imbalanced brightness.

For a better illustration, consider marker images (197 pixels × 193 pixels) acquired in the laboratory experiment shown in Figure 3. A typical way of using the marker for a displacement measurement is to find the centroid of the white circle surrounded by the black background, which can be obtained by image binarization. In case of the clear image shown in Figure 3a, the white circle is successfully separated from the background. On the contrary, when the image has imbalanced brightness (i.e., some background pixels are whiter than the circle), the binarization process fails to isolate the circle, resulting in an incorrect estimation of the centroid. As cameras are typically directed upward to focus on a structure where strong sunlight appears behind the structure in daytime, this light-related issue must be controlled.

Light-induced error must be carefully handled, as sunlight is inevitable in field testing. A strategy to overcome this issue is proposed in Section 3. The advantage of using the proposed strategy is experimentally verified in lab-scale and field testing environments in Section 4 and Section 5.

## 3. Displacement Measurement Using an Adaptive ROI

### 3.1. Adaptive ROI Algorithm

The proposed approach enables an accurate displacement measurement in a field-testing environment with adverse light exposure. To effectively address the light-induced feature detection error described in the previous section, this study proposes a computer vision-based displacement measurement strategy that focuses on a reliable feature detection algorithm with an adaptive ROI.

**Figure 4:**
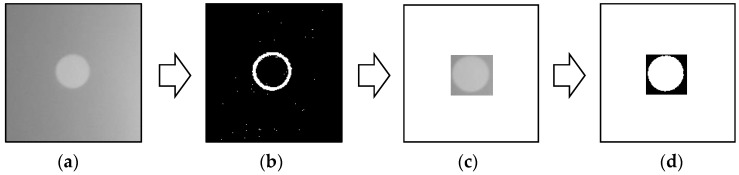
Flow of the adaptive region-of-interest (ROI) for the image in Figure 3b: (**a**) original image; (**b**) Sobel edge; (**c**) cropped ROI; (**d**) binarized image (Otsu threshold).

The adaptive ROI method is an automated and fast procedure to select the smallest ROI in each captured image. As shown in Figure 4, the adaptive ROI method is composed of four steps. The first step involves acquiring the boundary of the circle by applying an edge detection filter, such as the Sobel filter [56], to the original image. Typically, the filtered image has a hollow hole as shown in Figure 4b. This image is investigated to locate the smallest rectangular box that tightly contains the hollow hole, which is termed the adaptive ROI in this study. The image cropped by the adaptive ROI is shown in Figure 4c. Note that the cropped image contains a clear circle without a bright background, and this results in a clear distinction between the circle and the background. As shown in Figure 4d, the cropped image is binarized using a threshold method that helps in clearly separating the circle from the background. Finally, the centroid of the circle is calculated by averaging the location of the pixels in the circle. The overall procedure of the adaptive ROI takes 1.8 ms for a 200 pixels × 200 pixels size of an image with MABLAB, which can cover over 500 Hz of a feature detection process. Hence, the adaptive ROI method reliably detects features under adverse light conditions with sufficiently fast computation.

**Figure 5:**
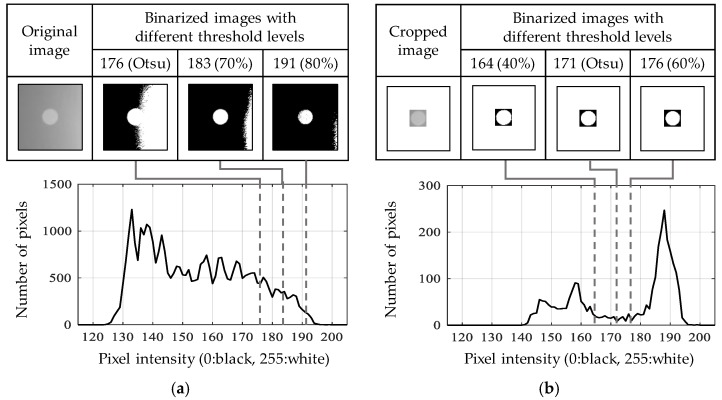
Histogram and the binarized image with different threshold levels for (**a**) original image; (**b**) cropped image along the adaptive ROI.

The adaptive ROI can handle the adverse light effect in the image. The original image with the whitened background shown in Figure 5a exhibits the histogram of the pixel intensity without clear separation between black and white. Thus, any threshold value, including the Otsu threshold as well as 70% and 80% of the pixel range, cannot fully isolate the circle from the background as shown in Figure 5a. On the contrary, the histogram of the cropped image by the adaptive ROI approach clearly has two groups of pixel intensities, each of which represents the circle and the background. Indeed, any threshold between the two groups can successfully binarize the cropped image. As such, the adaptive ROI provides a reliable means of feature detection tailored to a field-testing environment.

**Figure 6:**
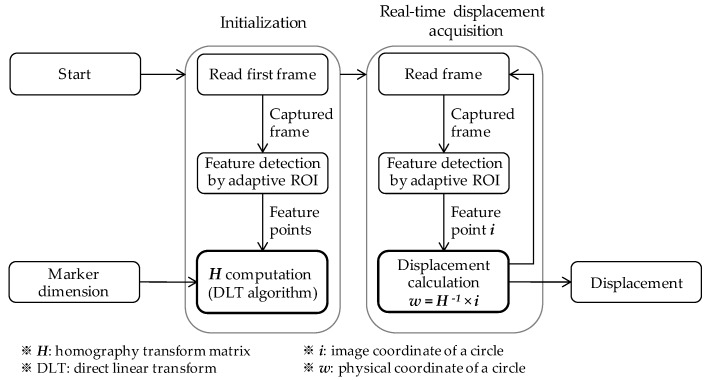
Flowchart of computer vision-based displacement measurement.

The flowchart in Figure 6 shows the overall process of the displacement measurement. The initialization step uses the feature positions (i.e., the centroids of the white circles) in the first image and the foreknown metric locations of the circles to compute the homography transform matrix. Here, the adaptive ROI method is employed in identifying the feature locations in the image to avoid the adverse effect of excessive exposure. The real-time displacement acquisition step commences when the direct linear transformation algorithm determines the homography matrix. The displacement acquisition step involves first detecting features with the adaptive ROI method from an incoming frame, and then using the homography transform to calculate the displacement. This process is repeated for each frame obtained from the camera.

### 3.2. Uncertainty Analysis

**Figure 7:**
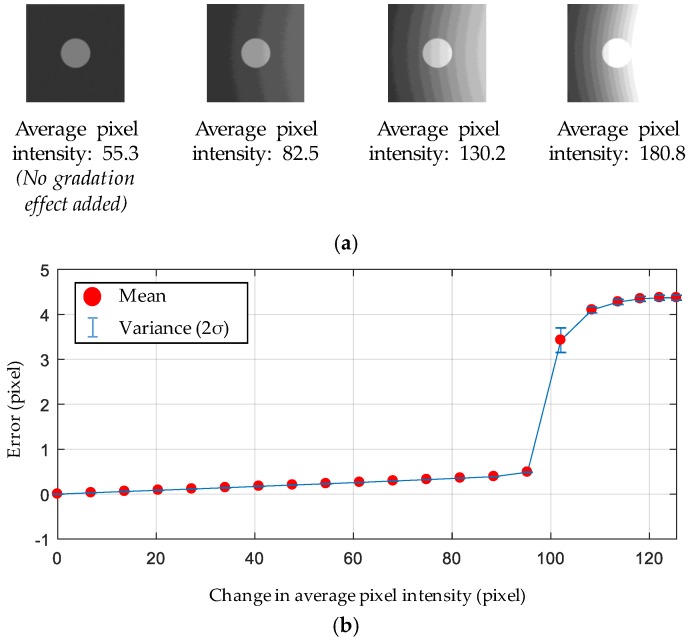
Error of the adaptive ROI method with respect to the light exposure. (**a**) Simulated marker images with added pixel intensity gradations; (**b**) error of the adaptive ROI method.

A numerical simulation for an uncertainty analysis was conducted to identify the effect of light on the accuracy of the adaptive ROI method. An image of 200 pixels by 200 pixels is numerically produced to have a clear white circle with a 30-pixel radius on a black background. Gaussian noise is added to the image for a realistic simulation. The added noise is assumed to be a zero-mean process with a variance of 1.8588, which is determined from a typical charge-coupled device (CCD) camera. The image’s degradation due to the excessive light exposure as shown in Figure 5a is simulated by adding a pixel intensity gradation so that the dark background on the right side of the image becomes lighter as shown in Figure 7. As a stronger pixel intensity gradation is added, the average pixel intensity of each image increases. Given an added gradation, the circle moves 0.01 pixel to the right until the circle completely moves 1 pixel, while the displacement estimation errors of the adaptive ROI method are calculated. The error is represented in terms of pixels, which can be readily converted to physical displacement values using a known scaling factor (mm/pixel). Figure 7b shows the error versus the average pixel intensity of each image. The drastic change in error around the average pixel intensity of 157 is caused when the white area expanded due to excessive light touching the circle in the center of the image. Thus, this is the limiting condition for the adaptive ROI method.

## 4. Experimental Validation: Laboratory-Scale

**Figure 8:**
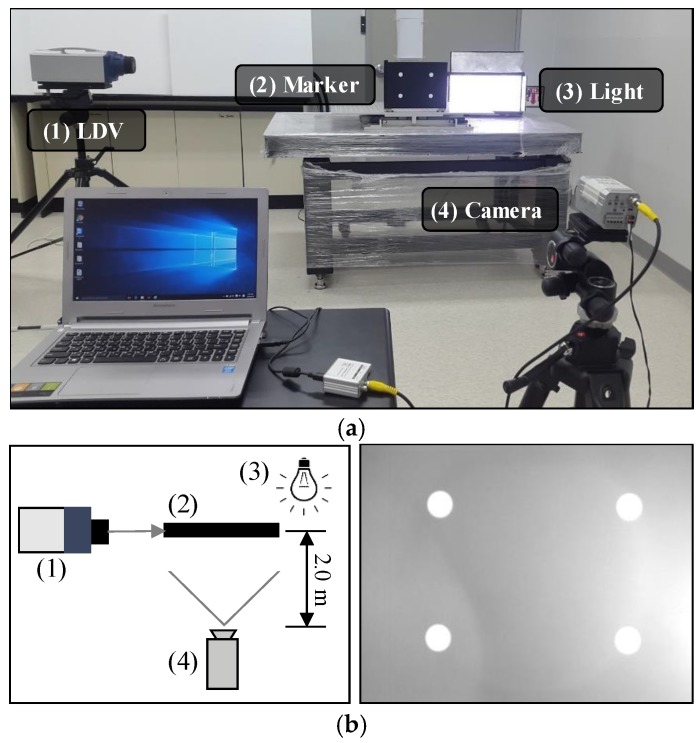
Laboratory-scale test: (**a**) experimental setup; (**b**) schematic view and captured frame. LDV: laser Doppler vibrometer.

A laboratory-scale experiment was conducted using a shaking table to investigate the robustness of the adaptive ROI method with respect to light-induced image degradation. Figure 8 illustrates the experimental setup, including the camera, target marker, computer, light source, and LDV. The camera is placed 2 m away from the target marker. The target marker is installed on a shaking table that provided harmonic excitation with a frequency of 1.5 Hz and an amplitude of 2 mm in the horizontal direction. An artificial light is placed behind the marker to produce image degradation. Here, the right side of the marker appeared to be light gray in the captured image due to the backlight, which makes the white feature circles indistinguishable from the background (see Figure 8b). The resulting displacements from the adaptive ROI are compared with the reference displacements measured by the laser Doppler vibrometer (LDV). The hardware configuration is summarized in Table 1.

**Figure 9:**
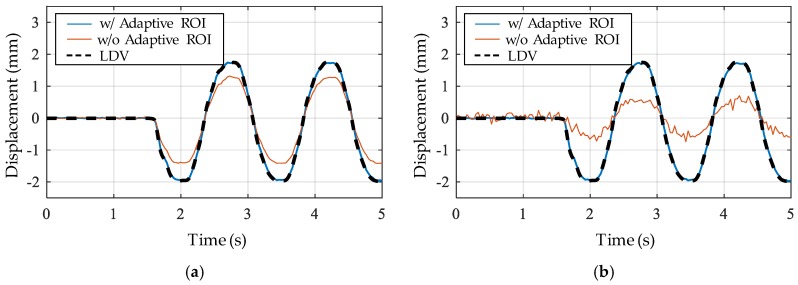
Comparison of the displacements with and without the adaptive ROI and by LDV from the feature circles at the (**a**) upper right; (**b**) upper left.

**Figure 10:**
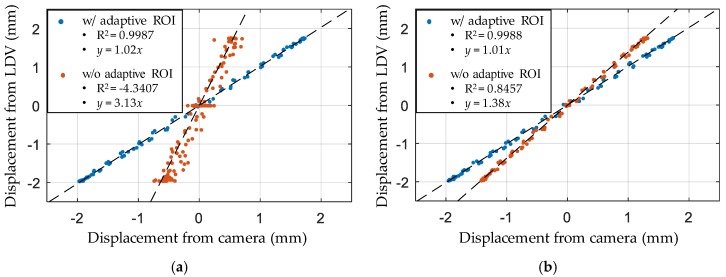
Correlation between the displacement from the camera and the LDV by a linear regression line from the circle at the (**a**) upper right; (**b**) upper left.

The displacement measured by the adaptive ROI was compared to those measured by the conventional approach (i.e., Otsu binarization without the adaptive ROI) and measured by LDV. The displacements calculated from the upper left and upper right circles are individually shown in Figure 9 to clearly demonstrate the light-induced errors. All displacements calculated using the adaptive ROI are accurately measured and agreed well with those from the LDV, even when the light significantly affected the marker images. However, the conventional approach, without the adaptive ROI, involved considerable errors, particularly with respect to the circles on the right, as expected from the image degradation caused by the right side of the background. Furthermore, the displacement from the left circles exhibits considerable errors, as shown in Figure 9b, because the centroid detection failure on the right circles directly leads to an erroneous homography matrix. For further error analysis, correlations between the displacements measured by the camera and the LDV are shown in Figure 10, which also confirms the advantage of using the adaptive ROI. The regression line in the case of the adaptive ROI has a coefficient of determination, R^2^, of 0.9987 and 0.9988 for the upper right and upper left, respectively. Furthermore, amplitude-dependent errors are not observed in that the regression line and the correlation plot are consistently close to each other over the entire amplitude range. The adaptive ROI-based feature detection method is thus expected to prevent possible large measurement errors that could frequently occur due to sunlight in a field-testing environment.

**Figure 11:**
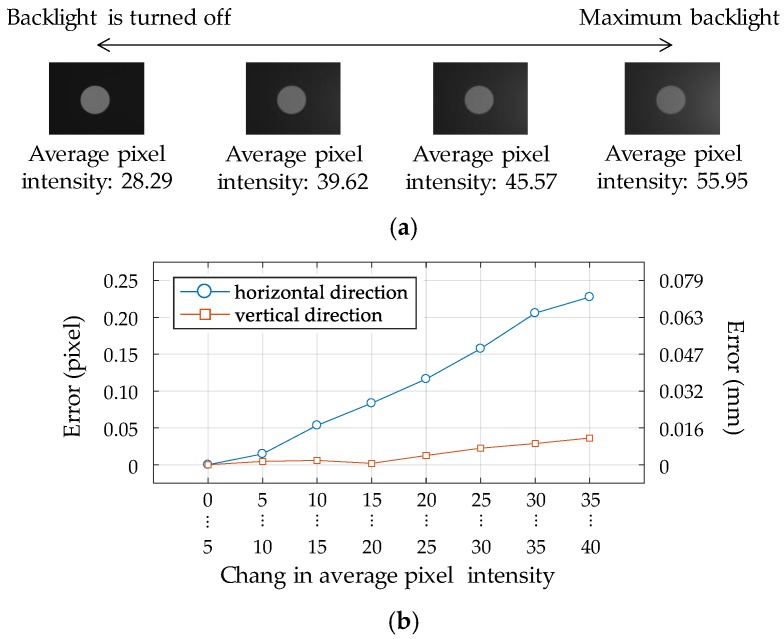
Measurement errors with varying light conditions. (**a**) Images with different backlights; (**b**) error for each direction.

The measurement uncertainty of the adaptive ROI method was experimentally examined with varying light conditions. Over 14,000 images were captured while the backlight located on the right side of the marker was gradually brightened to the maximum level of the lighting equipment as described in Figure 11a. The average pixel intensities of the marker images were increasing with stronger backlight. The trend of the measurement error is shown in Figure 11b in terms of the average pixel intensity. The captured images are categorized into nine groups in terms of the change in pixel intensity compared to the without backlight case. The displacements of the circle for each group are then averaged to identify the trend of the measurement error. Herein, the position of the feature point when the backlight is turned off is assumed to be the ground truth. The measurement error is at most 0.23 pixels in the horizontal direction and 0.04 pixels in the vertical direction. The measurement error in the horizontal direction is much larger than that in the vertical direction, because the backlight is placed on the right side of the marker in this experiment. The adaptive ROI method is observed to achieve sub-pixel accuracy even with an adverse light condition, which can produce large unacceptable errors unless properly handled.

## 5. Field Validation

**Figure 12:**
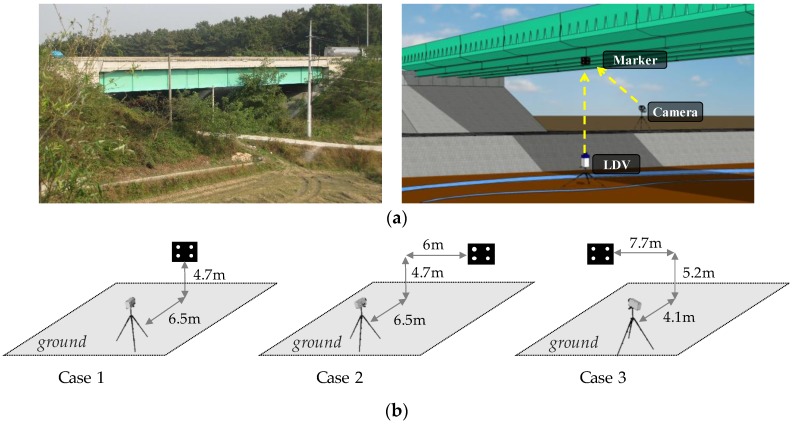
Experiment setup: (**a**) overview; (**b**) experimental cases with different camera locations to verify homography-based unconstrained camera positioning.

A full-scale experiment was performed at the Samseung Bridge, which is a 40-m long steel composite girder bridge located in Korea as shown in Figure 12a. The Samseung Bridge has been built for bridge testing purposes, providing an ideal field-testing environment. The same target marker as that used in the laboratory-scale experiment is attached to the bottom of the bridge deck at the mid-span using magnetic bases. As shown in Figure 12b, three different camera locations are considered to verify the benefit of the homography-based coordinate transform. An LDV that is located right below the target marker provides reference displacements to compare with those obtained from the camera. The LDV could be installed at the desired position because the area below the bridge involved an open space without any significant obstacles. This is not always the case with respect to most other bridges. A 29-ton truck operating on the bridge is used as an external load to produce bridge deflections.

**Figure 13:**
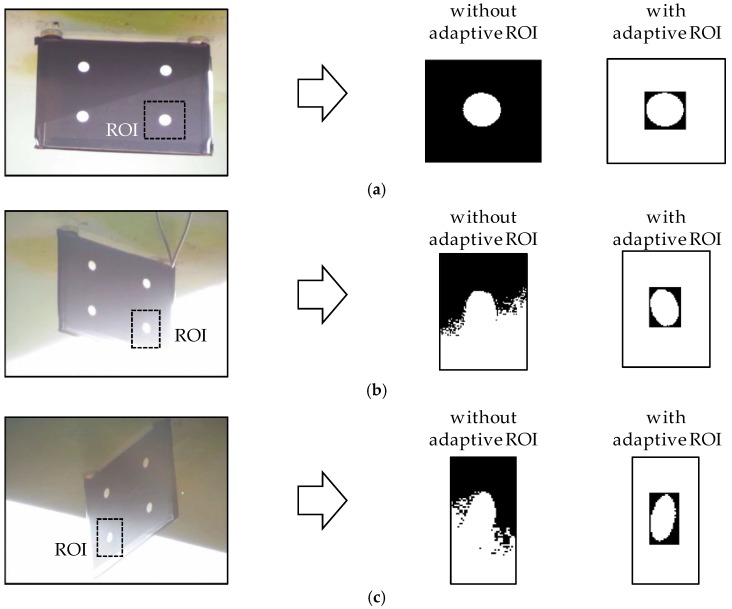
Feature detection with and without the adaptive ROI process: (**a**) Case 1; (**b**) Case 2; (**c**) Case 3.

As the experiment was conducted during the daytime, the captured images were observed to be affected by the sunlight and bright background as shown in Figure 13. The light from the sky severely changed the background brightness at the corner of the target marker. The degradation due to the excessive exposure impairs feature detection by the conventional approach, whereas the feature circles are correctly determined by using the adaptive ROI process in all three cases.

**Figure 14:**
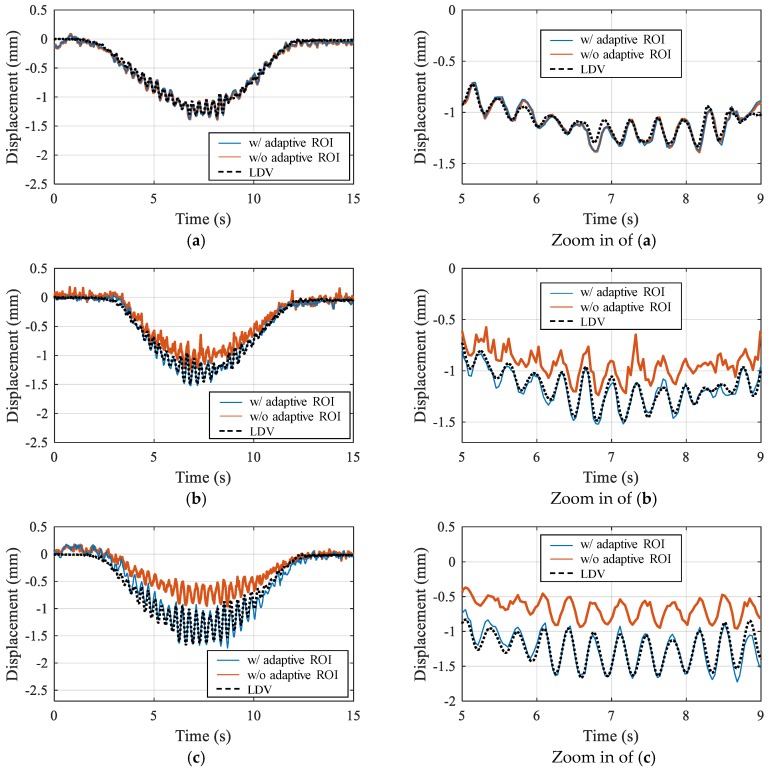
Comparison of measured displacements: (**a**) Case 1; (**b**) Case 2; (**c**) Case 3.

The measured displacements calculated by the proposed method are compared with those obtained by the conventional technique without the adaptive ROI as well as the references from the LDV. In all three cases, as shown in Figure 14, the displacements calculated with the adaptive ROI and the homography transform are consistently close to those measured by the LDV. In the case without the adaptive ROI, the feature detection failure that occurred in Cases 2 and 3 results in significant errors in the calculated displacement. For a quantitative demonstration of the results, two error indicators are defined as:

(2) $$E_{\max}=\left|\max\left(\left|u_{camera}\right|\right)-\max\left(\left|u_{exact}\right|\right)\right|$$

(3) $$E_{sd}=\left|\frac{\sigma_{camera}-\sigma_{exact}}{\sigma_{exact}}\right|$$

where |∙| denotes the absolute value, and ***u****_camera_* and ***u****_exact_* are the displacement measured by the camera and LDV, respectively. **σ***_camera_* and **σ***_exact_* are the standard deviations of the displacement from the camera and LDV, respectively. The error indicators calculated for each case are summarized in Table 2, and verify the observations in Figure 14. Thus, the results indicate that the proposed computer vision-based approach provides accurate displacement measurements with robustness to unfavorable light conditions and flexibility in camera position.

**Figure 15:**
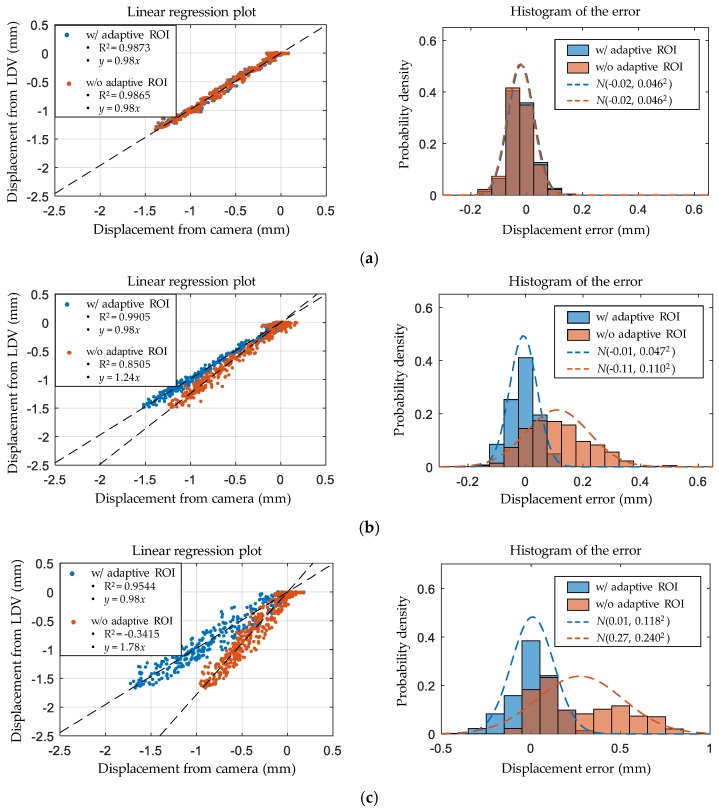
Error analysis by means of a linear regression model and a histogram of the error for (**a**) Case 1; (**b**) Case 2; (**c**) Case 3.

In addition to the error measures, the correlation between the displacements from the camera and the LDV is shown in Figure 15 to further analyze the error characteristics. Case 1 with the clear marker has a regression line with a slope of 1 and R^2^ of 0.9873 when the adaptive ROI is used. For Cases 2 and 3, the correlation plots deviate more from the regression lines because of the strong backlights, resulting in lower R^2^; however, the adaptive ROI can successfully correct the slope of the regression line to 1. This observation also can be verified from the error histograms in Figure 15. The error distributions of the adaptive ROI have mean values close to zero with smaller standard deviations compared to the displacement obtained without the adaptive ROI. Thus, the adaptive ROI can effectively handle the adverse effect of light on recording the marker image.

## 6. Conclusions

In the present study, a reliable computer vision-based approach to provide a practical means for structural displacement measurement was presented. To maximize the applicability of the vision-based system to full-scale civil engineering structures, the proposed approach focused on addressing image degradation due to excessive exposure. To this end, an adaptive ROI process was developed to reliably detect the features on the target marker even when undesired light significantly affects the captured maker image.

The proposed structural displacement measurement method was validated in both laboratory and field-testing environments. A laboratory-scale experiment with artificial light to generate the image degradation was conducted. Three field experiments were subsequently conducted at the Samseung Bridge to validate the performance of the adaptive ROI method. In addition, the experiments also verified the unconstrained camera positioning provided by the homography transform in the field testing environment. The images of the feature circles on the target marker were significantly illuminated, as the camera was on the side looking up the bridge. This condition was expected to be common in this type of field experiment. The proposed method measured the displacement with subpixel accuracy even with the light-induced image degradation. The results also indicated that the proposed method reliably tracked the displacements of the target marker at three different camera locations using the homography transform. In conclusion, the structural displacement measurement method examined in the present study is reliable as well as suitable for field applications which require robustness to adverse light and enhanced flexibility in selecting camera locations.

## Conflicts of Interests

The authors declare no conflict of interest.

## Figures and Tables

**Table 1:** Hardware specifications.

Parts	Model	Features
Marker	*User-defined*	- Four white circles in a black background
		- Horizontal interval: 150 mm
		- Vertical interval: 100 mm
		- Radius of the circles: 10 mm
Camera	*CNB-A1263NL*	- NTSC output interface ^1^
		- ×22 optical zoom
Computer	*LG-A510*	- 1.73 GHz Intel Core i7 CPU
		- 4 GB DDR3 RAM
LDV	*RSV-150*	- Displacement resolution: 0.3 μm

^1^ 640 × 480 resolution at 29.97 fps. CPU: central processing unit; RAM: random access memory; NTSC: national television system committee.

**Table 2:** Measurement errors.

Cases	Feature Detection	*E_max_*	*E_sd_*
Case 1	without adaptive ROI	0.0549 mm	0.0185
	with adaptive ROI	0.0433 mm	0.0126
Case 2	without adaptive ROI	0.2518 mm	0.1736
	with adaptive ROI	0.0314 mm	0.0127
Case 3	without adaptive ROI	0.7110 mm	0.4081
	with adaptive ROI	0.0565 mm	0.0439
